# Lack of Evidence for a Role of ACE-2 Polymorphisms as a Bedside Clinical Prognostic Marker of COVID-19

**DOI:** 10.3390/v15071448

**Published:** 2023-06-27

**Authors:** Josè R. Fiore, Mariantonietta Di Stefano, Andrew Oler, Yu Zhang, Jingwen Gu, Clifton L. Dalgard, Giuseppina Faleo, Brian Epling, Luigi Notarangelo, Andrea Lisco, Teresa A. Santantonio

**Affiliations:** 1 Infectious Diseases Unit, Department of Medical and Surgical Sciences, University of Foggia, 71122 Foggia, Italy; mariantonietta.distefano@unifg.it (M.D.S.); giuseppina.faleo@unifg.it (G.F.); teresa.santantonio@unifg.it (T.A.S.); 2 Bioinformatics and Computational Biosciences Branch, Office of Cyber Infrastructure and Computational Biology, National Institute of Allergy and Infectious Diseases (NIAID), National Institutes of Health, Bethesda, MD 20892, USA; andrew.oler@nih.gov (A.O.); jingwen.gu@nih.gov (J.G.); 3 Immune Deficiency Genetics Disease Section, Laboratory of Clinical Immunology and Microbiology, National Institute of Allergy and Infectious Disease (NIAID), Division of Intramural Research (DIR), National Institutes of Health, Bethesda, MD 20892, USA; yu.zhang@nih.gov (Y.Z.); luigi.notarangelo@nih.gov (L.N.); 4 Collaborative Health Initiative Research Program, The American Genome Center, Uniformed Services University of the Health Sciences, Bethesda, MD 20892, USA; 5 Department of Anatomy, Physiology & Genetics, Uniformed Services University of the Health Sciences, Bethesda, MD 20892, USA; 6 National Institute of Allergy and Infectious Diseases (NIAID), National Institutes of Health, Bethesda, MD 20892, USAandrea.lisco@nih.gov (A.L.)

**Keywords:** severe acute respiratory syndrome coronavirus-2 (SARS-CoV-2), angiotensin-converting enzyme 2 (ACE-2), respiratory distress syndrome (ARDS), single nucleotide polymorphism (SNP)

## Abstract

The novel SARS-CoV-2 coronavirus causes a severe respiratory syndrome referred to as coronavirus disease (COVID-19). The angiotensin-converting enzyme 2 (ACE-2) plays an important role as a cellular receptor for SARS-CoV-2 and is largely expressed in lungs, kidneys, heart and the gastrointestinal tract along with being shed in plasma. The ACE-2 gene and protein show a high level of genetic polymorphism, including simple nucleotide variation, transcriptional variation, post-transcriptional changes, and putative protein mutations that could interfere with the binding or entry of SARS-CoV-2 and affect tissue damage in lungs or other organs. Genetic polymorphisms can impact SARS-CoV-2 viral entry and COVID-19 severity. This single-center study evaluated the possible role of the main ACE-2 polymorphisms (rs143936283, rs2285666, rs41303171, rs35803318, and rs2106809) as potential prognostic markers in SARS-CoV-2-infected individuals. Frozen whole blood was used for DNA isolation and genomic DNA samples were sheared using the Covaris LE220 Focused-ultrasonicator for targeting a peak size of 410 bp. Whole-genome sequencing libraries were generated from fragmented DNA using the Illumina TruSeq DNA PCR-Free HT Library Preparation Kit and sequenced on an Illumina NovaSeq 6000. We did not identify any correlation between ACE-2 polymorphisms and COVID-19 prognosis, suggesting that the interpretation and clinical use of ACE-2 genetic polymorphisms in real-world clinical settings requires further experimental and clinical validation.

## 1. Introduction

The Severe Acute Respiratory Syndrome Coronavirus-2 (SARS-CoV-2) is a novel Coronavirus [1] so named because of the crown-like spike protein (S) present on the viral envelope. The genome contains approximately 30,000 nucleotides and is phylogenetically included in the Betacoronavirus genus, which includes two other zoonotic coronaviruses (the severe acute respiratory syndrome coronavirus (SARS-CoV) and the Middle East respiratory syndrome coronavirus (MERS-CoV)), as well as other less pathogenic coronaviruses (HCoV-OC43, HCoV-HKU, HCoV-229E, and HCoV-NL63), which are generally associated with mild and self-limiting upper respiratory tract infections [2].

SARS-CoV-2 is the etiological agent of COVID-19, a severe and progressive upper and lower respiratory tract infection that emerged in the Huanan Seafood and Animal Market in the city of Wuhan, located in the Hubei province of China, in early December 2019. The rapid global spread of COVID-19 represents the most significant pandemic of the 21st century to date, causing 761,071,826 confirmed cases and 6,879,677 deaths by March 2023.

SARS-CoV-2 is efficiently transmitted by exposure to respiratory droplets with coughs or sneezes from infected patients or in the form of fomites on surfaces [3]. SARS-CoV-2 is responsible for a broad spectrum of clinical manifestations ranging from asymptomatic or influenza-like symptoms (fever, dry cough, mild dyspnea, rhinitis, myalgia, fatigue) to severe viral pneumonia, acute respiratory distress syndrome (ARDS), respiratory failure, and death [4]. COVID-19 may also be associated with neurological manifestations such as ageusia and anosmia, which can appear before respiratory symptoms, as well as headache, delirium, and epileptic seizures. 

It is well-documented that angiotensin-converting enzyme 2 (ACE-2) is the SARS-CoV-2 host entry receptor into respiratory epithelial cells [5]. ACE-2 is a carboxypeptidase with several known physiological functions, including regulation of blood pressure and water balance in the Renin-Angiotensin-Aldosteron system (RAAS). ACE-2 was discovered in 2000 as a homologue of the ACE protein with distinct catalytic function on angiotensins and a non-redundant role in the RAAS system. ACE-2 had already been identified as a SARS-CoV receptor, but the affinity of ACE-2 for the SARS-CoV-2 spike glycoprotein is 10- to 20-fold higher than that of SARS-CoV. Therefore, the determinant of SARS-CoV-2 tropism is the spike glycoprotein, which forms trimers on the virion surface [6,7,8,9]. The spike protein has two subunits: the S1 subunit, which mediates binding to the ACE-2 receptor, and the S2 subunit, which is responsible for membrane fusion. Virion entry into the respiratory epithelium is a complex process that implicates ACE-2 binding, cleavage of the spike subunit S1 and S2 by the host transmembrane serine protease TMPRSS2, and eventually host and viral membrane fusion with consequent release of viral RNA in the host cell. Functional interference with ACE-2 and altered homeostatic regulation of ACE and ACE-2 functions have been proposed as a possible risk modifier of COVID-19 clinical progression [10]. 

The heterogeneity in the severity of illness among COVID-19 patients suggests that host genetics could account for such inter-individual variability. 

As SARS-CoV-2 primarily depends on ACE-2 for fusion and entry, ACE-2 variation might be considered one of the reasons for these clinical outcome differences.

As a matter of fact, several studies inferred that ACE-2 genetic polymorphisms or rare variants may influence individual susceptibility to the disease and severity of illness [11,12,13,14,15,16,17].

Structural modeling of exonic polymorphisms predicted increased or decreased binding to the spike protein in silico or in vitro [16], which could increase or decrease the susceptibility to viral entry or pathogenesis. While not experimentally tested, intronic polymorphisms could affect mRNA splicing and the relative and absolute distribution of different transcripts.

These observations led to the proposal that ACE-2 polymorphisms might serve as a possible prognostic marker of COVID-19 clinical course or response to immunomodulatory treatments [5,11,12,13,14,15,16,17]. Several ACE-2 polymorphisms were then claimed to have a prognostic value in SARS-CoV-2 infection in different studies and populations, and some specific single nucleotide polymorphisms (SNPs) were more commonly studied: rs143936283, rs2285666, rs41303171, and rs35803318. However, aside from in silico and epidemiological studies suggesting that ACE-2 variants could impact cell binding-entry events or the increase in quantitative ACE-2 expression (thus influencing prognosis), the clinical implication of such genetic variability is still not fully established [17,18,19].

Here, we report the results of a single-center study on the prevalence of ACE-2 polymorphisms in a small cohort of SARS-CoV-2-infected patients with various clinical phenotypes of COVID-19, aiming to evaluate the potential utility of these polymorphisms as prognostic markers in routine clinical practice.

## 2. Materials and Methods

### 2.1. Study Participants and Sample Collection

We retrieved deidentified whole blood from leftover samples collected for clinical indications from adult patients sequentially admitted to the Clinic of Infectious Diseases, Foggia University Hospital, Foggia (Italy), during the first wave of SARS-CoV-2 infections between February and September 2020. All patients had molecular confirmation of SARS-CoV-2 infection by polymerase chain reaction (PCR) on nasopharyngeal samples. All leftover samples were collected near the time of hospital admission and before any medical treatment. At the time of this study, antivirals such as remdesivir and monoclonal antibodies were unavailable. Deidentified clinical and laboratory data were recorded for all patients. Disease severity was categorized as mild-moderate or severe-critical based on the Diagnosis and Treatment Protocol for Novel Coronavirus pneumonia (trial version 7) [20]. Briefly, cases were designated as: mild—minimal symptoms, no imaging findings; moderate—fever, respiratory symptoms, and radiographic pneumonia; severe—respiratory distress (≥30 breaths/min) or oxygen saturation ≤93% at rest; or critical—shock, ARDS, respiratory failure, or other organ failure requiring intensive care.

### 2.2. DNA Isolation, Sequencing, and Variant Analysis

Frozen whole blood was used for DNA isolation using the QIAsymphony DNA Midi Kit (Qiagen, Hulsterweg 82, Venlo, The Netherlands) and genomic DNA samples were sheared using the Covaris LE220 Focused-ultrasonicator for targeting a peak size of 410 bp. Whole-genome sequencing libraries were generated from fragmented DNA using the Illumina TruSeq DNA PCR-Free HT Library Preparation Kit (5200 Illumina way San Diego, CA 92122, USA) and sequenced on a current Illumina NovaSeq 6000 platform using an S4 Reagent Kit using 151 + 8 + 8 + 151 cycle run parameters. Variant calling was performed as previously reported [21]. Variants were normalized and decomposed using vt (v.0.5772), annotated using a variant effect predictor [21], and filtered using gemini (v.0.30.2). Variants in and around the ACE-2 gene, including 5 kb upstream and downstream, were selected based on specific criteria. These criteria required the variants to pass the quality filter, have a minimum read depth of 8 bp, minimum genotype quality score of 20, and a minimum allelic balance of 25%. Data for all patients have been deposited under dbGaP accession number phs002245 as a subset.

## 3. Results

Thirty-nine SARS-CoV-2-infected patients consecutively admitted to the Clinic of Infectious Diseases, Ospedali Riuniti University Hospital, Foggia (southeastern Italy) were enrolled in the present study.

Nineteen patients were males, and twenty were females. The mean age was 56.4 years (ranging from 23 to 98 years). Of the 39 patients, 6 (15%) had severe/critical disease characterized by severe pneumonia (ARDS syndrome in 4 cases), 28 displayed moderate or mild disease with clinical and/or radiological findings of pneumonia but without hypoxia or respiratory distress, and 5 were clinically asymptomatic.

Pre-existing co-morbidities included hypertension (19 cases), chronic bronchitis (7 cases), diabetes (11 cases), and chronic hepatitis (3 cases).

The genomic variations of the ACE-2 receptor gene among the 39 SARS-CoV-2-infected patients were analyzed by whole-genome sequencing (WGS). Seventy-one SNPs were located in intronic regions, eight were found in the 3`-prime downstream-gene region, five in the 5`-prime upstream-gene region, one was located in a splice region (rs2285666), and one polymorphism was found in an exonic region coding for the transmembrane portion of the protein (rs35803318).

We concentrated our analysis on SNPs that were analyzed twice or more in different studies, namely, rs143936283, rs2285666, rs41303171, and rs35803318. In addition, we considered rs2106809, which was recently studied in a clinical report [18].

The prevalence of ACE-2 intronic variants rs2285666 and rs2106809 was 30.7% for each of these. The SNVs were equally distributed by gender (rs2285666 was in six males and six females; rs2106809 was in five males and seven females) and were not associated with severity of COVID-19 disease (Table 1).

The SNP rs35803318 was observed in only one patient (2.5%), whereas rs41303171 and rs14393628 were not identified in any patient enrolled in this study.

## 4. Discussion

COVID-19 has a variable clinical course which can only in part be explained by age, gender, or comorbidities. Accordingly, there has been extensive research into clinical or laboratory predictors that could improve prognostic assessment and guide the use of immunomodulant or antiviral treatments.

Since host receptors are essential for viral entry and pathogenesis, changes in the level of expression, sequence, and function of such host viral receptors can, to some extent, affect the clinical course of viral diseases. The role of the host receptor’s genetic variation in viral diseases has been extensively studied in the HIV infection model; in particular, the CCR5 co-receptor Δ32 deletion can prevent HIV infection in homozygous individuals carrying such a genotype [22] and lead to the cure of HIV-1 infection in subjects who undergo conditioning and hematopoietic stem cell transplantation with a CCR5 Δ32 homozygous donor [23]. Similarly, among the Coronaviruses, the MERS-CoV spike glycoprotein requires the host cell type II dipeptidyl peptidase 4 (DPP4/CD26) for viral entry. It was reported that some DPP4 polymorphisms (K267E, K267N, A291P, and Δ346–348) can strongly reduce the spike binding and consequent viral entry of MERS-CoV into target cells, resulting in reduced viral replication [24].

In SARS-CoV, and similarly to SARS-CoV-2, the S1 domain of the spike protein mediates binding to the ACE-2 receptor. The viral receptor binding domain (RBD) responsible for binding to ACE-2 has been mapped within S1 between amino acids 318 and 510; the S1 position between 424 and 494 mediates direct contact with the first α-helix, the Lys in position 353, and the proximal residues at the N-terminus of β-sheet 5 of ACE-2. By modifying the amino acid His 353 in rat ACE-2 and modifying an additional site (Asp 90) that may alter the conformation of the α-helix 1 of ACE-2, rat ACE-2 can be converted into an efficient entry receptor for SARS-CoV, providing access to a robust animal model for pathogenesis studies. Similarly, a point mutation, Leu584Ala in ACE-2, can markedly facilitate the entry of SARS-CoV into target cells [13].

Therefore, while some in vitro and animal model data corroborate the role of ACE-2 as the host receptor for SARS-CoV-2, the role of polymorphisms and genetic variants of ACE-2 as possible prognostic markers in COVID-19 has been proposed. In fact, ACE-2 is largely expressed in many cell types of the respiratory tract—the main route of virus entry to the body—as well as in the cardiovascular system, where additional important clinical manifestations of COVID-19 frequently occur [12,13,14,15].

ACE-2 is an interferon-regulated gene containing 20 introns and 18 exons located on chromosome Xp22 [9]. It is expressed as different transcripts in a wide range of organs/tissues in the human body; in addition to the respiratory epithelium and endothelial cells, it is also expressed in the gastrointestinal tract, kidney, skin, male testis, female breast, and in various CNS cells, such as neurons, astrocytes, oligodendrocytes, and microglial cells [11]. Accordingly, autopsy studies identified viral genetic material in several tissues of infected individuals with fatal infections [25].

The ACE-2 gene and protein show a high degree of genetic polymorphisms with racial and ethnic variation, resulting in functional implications on the RAAS pathway [12]. Such variants include single nucleotide mutations, transcriptional variation, post-transcriptional modifications, and putative protein mutations [13]. The single nucleotide polymorphisms (SNPs) have made their way into the scientific spotlight; recently, Suryamohan and collaborators found 298 unique protein-altering variants across 256 codons distributed throughout the 805 amino acids of human ACE-2 (hACE-2) [14].

ACE-2 gene polymorphisms (SNPs) have been associated with several lung diseases, such as chronic obstructive pulmonary disease (COPD), pulmonary hypertension, asthma, acute lung injury, ARDS, lung cancer, and pulmonary sarcoidosis [15]. Similarly, ACE-2 polymorphisms have also been associated with heart diseases, hypertension, and renal failure [26].

As for these other clinical contexts, the role of ACE-2 host genetics has been proposed to play a possible role in the interindividual variability of COVID-19 clinical severity.

Some studies, in fact, suggest that polymorphisms in ACE-2 could be related either to susceptibility to the virus or to the severity of clinical manifestations of the disease, although other studies have failed to corroborate these findings [11,12,13,14,15,16,17].

In this study, we evaluated the possible association between ACE-2 variants and COVID-19 clinical outcomes in patients admitted to a single tertiary-level University Hospital to define the possible use of these biological and genetic data as a prognostic marker in daily clinical practice.

To this purpose, we correlated the presence of ACE-2 SNPs previously proposed as possible prognostic factors with the clinical outcomes of patients of our cohort. We did not identify any significant differences in the severity of disease and clinical outcomes in patients carrying any of the analyzed SNPs.

Our findings are in agreement with other recent reports where the role of ACE-2 polymorphisms as a prognostic marker of SARS-CoV-2 infection was not corroborated [17,18,19].

It is conceivable that the interaction between clinical predictors (i.e., ethnicity, gender, age, and comorbidities) and genetic predisposing factors in COVID-19 may be quite complex and intricate and, thus, may require large prospective studies to be elucidated. In fact, a limitation of our study is its limited power. Alternatively, despite the role of ACE-2 in host cell entry, the cellular transmembrane protease serine 2 (TMPRSS2) could further affect the interaction between the viral spike, ACE-2, and viral entry and replication [27]. Variants in other genes involved in IFN signaling and more recently in lung function (i.e., MUC5B and SFTPD) SNPs were found to be related to infection severity [11,12].

In addition, one should consider that an alternative (ACE-2-independent) receptor for the entry of SARS-CoV-2 into cells has been proposed (i.e., CD147, the extracellular matrix metalloproteinase inducer (EMMPRIN)) that could also impact clinical outcomes [28].

It is important to mention that clinical predictors and genetic, epigenetic, and viral co-factors interact in complex models and that this may render the interpretation of each isolated variable in the acute clinical setting in some clinical contexts to be very difficult.

In addition, many other genetic and/or behavioral factors, such as the genes related to innate and adaptive immunity, viral load, and the preventive precautions taken at individual and country levels may also influence COVID-19 virulence and modify disease outcomes.

Furthermore, not only must genetic factors be taken into account to explain the differences in the outcomes of COVID-19, but also environmental risk factors such as smoking, drinking habits, and environmental pollutants.

An important limitation of this study that we must recognize is the small sample size of our patient series and the consequent low statistical power of our analysis. With a significance level of 0.05, the power to identify an association of the common variants rs2285666 and rs2106809 with COVID-19 clinical features was 0.279, which is suboptimal.

Nevertheless, we meant to evaluate the potential usefulness of such genetic analyses in daily real-life clinical practice. The results presented here indicate that although a general genetic influence in SARS-CoV-2 vulnerability is quite probable, we still do not have sufficient evidence at this time to support the use of ACE-2 SNPs as a prognostic tool for risk stratification in clinical practice. Further studies on other epigenetic factors must evaluate the potential impact of interindividual genetic variability on COVID-19 clinical outcomes. So much is yet to be known.

## Figures and Tables

**Table 1:** *ACE-2* polymorphism detection in a group of SARS-CoV-2-infected subjects.

Polymorphism Detection		Asymptomatic 5	Mild/Moderate 28	Severe/ARDS 6
Reference/Alternative				
rs2285666	C/T	0	10 (35.7%)	2 (33.3%)
rs2106809	A/G	0	10 (35.7%)	2 (33.3%)
rs3503318	C/T	0	1 (3.6%)	0
rs41303171	T/C	0	0	0
rs143936283	T/C	0	0	0

Legend: number of patients by different clinical conditions with ACE-2 polymorphisms in the study group.

## Data Availability

The data of the study are available on request.
